# Editorial: Exploring prevention strategies and treatment in addictive disorders

**DOI:** 10.3389/fpsyt.2024.1432822

**Published:** 2024-07-04

**Authors:** Sandra Montagud-Romero, Victoria Gómez-Murcia, Francisco José Fernández-Gómez, Cristina Núñez

**Affiliations:** ^1^ Department of Psychobiology, Facultad de Psicología, Universitat de València, Valencia, Spain; ^2^ Group of Cellular and Molecular Pharmacology, Department of Pharmacology, University of Murcia, Murcia, Spain; ^3^ Instituto Murciano de Investigación Biosanitaria (IMIB) – Pascual Parrilla, Murcia, Spain

**Keywords:** vulnerability factors in addictive disorders, therapeutic approaches for addictive disorders, genetics and epigenetics of addictive disorders, pharmacological targets for addictive disorders, substance use disorders (SUD), cognitive-behavioral therapy and addictive disorders, gaming disorder (GD)

Addictive disorders are characterized as chronic illnesses that affect brain circuits related to reward, motivation, and memory. Dysfunctions in these circuits are associated with typical Substance Use Disorders (SUDs) features, such as the inability to abstain from seeking and using drugs, uncontrollable cravings, as well as impaired interpersonal relationships (1). Relapse is a key factor in SUDs, as a significant number of individuals who undergo treatment experience periods of relapse. This phenomenon underscores the chronic and complex nature of SUDs, where sustained abstinence is often challenging to achieve. Various factors contribute to relapse, including environmental triggers, stress, emotional instability, social pressure, and untreated mental health issues. These factors can interact and create a cycle wherein individuals struggle to maintain sobriety despite initial treatment efforts. Understanding the dynamics of relapse is crucial for developing effective interventions and support systems that address the multifaceted challenges of SUDs recovery. Despite extensive research on the neurocircuits implicated in addictive disorders over several decades, vulnerability factors and the neuronal, cellular, and molecular pathways underpinning these mechanisms remain inadequately understood (2).

In order to uncover new therapeutic approaches to obtain suitable treatments for SUDs, several strategies are being considered. The properties of pentilludin, a new and promising drug whose target is the receptor type protein tyrosine phosphatase D, are revised in this Research Topic (Uhl). The efficiency of the available pharmacotherapy to treat SUDs may vary from one abused drug to another. The study of Wang et al. revealed that buprenorphine, which is currently used to treat opioids and cocaine use disorders, could be a promising drug also for methamphetamine use disorder and relapse. Furthermore, the possible similarities between attention-deficit/hyperactivity disorder and SUDs have led van Ruitenbeek et al. to propose methylphenidate, which is indicated for attention-deficit hyperactivity disorder, as a potential effective drug as well for SUDs.

Besides pharmacological interventions, cognitive-behavioral therapy (CBT) has been proposed as an alternative approach for treating SUDs, together with pharmacotherapy or alone (3). In this Research Topic, it is reported the efficacy of a self-directed treatment workbook based on CBT and one motivational interview to promote changes in cannabis use in individuals who wish to recover with minimal professional support (Schluter et al.). Moreover, Hofsted et al. tested a 15-week CBT designed specifically for Gaming Disorder, that is recognized by the International Classification of Diseases under the section for addiction, and found statistically significant decreased symptoms, reduction in gaming hours and an increase in non-gaming leisure activities with reductions in depression and anxiety. On the other hand, parental monitoring has been proposed as a useful tool to protect adolescents against SUDs. In the study performed by Alexander et al. this hypothesis was examined and, through the twin design, genetic and environmental contributions to these relationships were measured. Although genetic influences on substance use and parental monitoring were detected, the data of this investigation point out to limited causal connection between parental monitoring and substance use in mid-to-late adolescent community samples.

The global rise in SUDs poses a significant health challenge driven by complex neurobiological mechanisms and exacerbated by lifestyle stressors. Understanding the molecular pathways and vulnerability factors underlying SUDs stages is essential for developing effective treatments. Thus, Mu et al., through a cross-sectional study, assessed the psychological factors and the history of methamphetamine use that could affect methamphetamine relapse. They found that individuals with methamphetamine use disorder informed of worse executive function and mental health. Their results also seem to indicate that the relapse rate might be influenced by the lower age of first methamphetamine use, while, specifically, repeated relapse could affect executive dysfunction.

Bioinformatics have become a crucial tool to elucidate different pathways involved in a same etiopathogenic origin. However, their results need to be examined in detail for a better interpretation. In this line, the role of the serine proteinase inhibitor A3 (SERPINA3), a differentially expressed gene identified by GEO2R tool, has been explored and identified as an important upregulated protein in alcohol use disorder, but it appears not usable as a predictive relapse marker (Zhang et al.).

Research has shown that microRNAs play a pivotal role in opioids abuse (4, 5). Concordantly, in this Topic Shi et al. report that the expression levels of miR-124 and its target protein IQGAP1 are linked with anxiety and depression symptoms, altering susceptibility and cognitive function in patients with morphine dependence.

Research efforts deal with a variety of abused substances as well as other addictive disorders, offering novel pharmacological targets and promising therapeutic approaches. However, further research is needed to establish evidence-based treatments, especially considering psychiatric comorbidities. Overall, the findings described in this Research Topic highlight the complexity of addictive disorders and the need for multifaceted approaches to its treatment and prevention.

## Author contributions

SM-R: Supervision, Writing – review & editing. VG-M: Writing – original draft. FF-G: Writing – original draft. CN: Supervision, Writing – original draft, Writing – review & editing.
